# Perioperative Management and Preemptive ECMO Cannulation of a Parturient with Cystic Fibrosis Undergoing Cesarean Delivery

**DOI:** 10.1155/2020/8814729

**Published:** 2020-12-04

**Authors:** Thais Franklin Dos Santos, Andrea Rabassa, Oscar Aljure, Reine Zbeidy

**Affiliations:** Department of Anesthesiology, University of Miami, Jackson Memorial Hospital, 1611 NW 12th Ave, Miami 33136, FL, USA

## Abstract

Physiologic changes of pregnancy and cystic fibrosis pathology provide a unique set of circumstances. Pulmonary disease accounts for over 90% of the morbidity and mortality of patients with cystic fibrosis. These abnormalities create anesthetic challenges due to multiple organ systems being affected including the respiratory, gastrointestinal, cardiovascular, and genitourinary tracts, where patients present with chronic respiratory failure, pancreatic insufficiency, poor nutrition, and cardiac manifestations. We present the perianesthetic management of a parturient with cystic fibrosis who successfully underwent preterm cesarean delivery under neuraxial anesthesia with preemptive bilateral femoral venous sheaths placed for potential extracorporeal membrane oxygenation (ECMO) initiation.

## 1. Introduction

Cystic fibrosis (CF) is an autosomal recessive disease that currently affects at least 30,000 people in the United States [1]. Manifestations include severe pulmonary compromise, pancreatic insufficiency, diabetes mellitus, and poor nutrition, leading to increased mortality. Since the 1930s when CF was first described, advances in preventative measures and treatment significantly improved the poor prognosis and increased these patients' life expectancies from a median of 5 years to around 30 years old [1]. As a result, more women survive to an age where they can consider starting their own families [2].

The normal physiologic changes of pregnancy and CF pathology provide a unique set of circumstances. In CF, patients inherit a mutation on the long arm of chromosome 7 that encodes a chloride channel called the CF transmembrane regulator (CFTR). Defective CFTRs lead to a buildup of viscous secretions and pathologic changes to multiple organ systems including the respiratory, gastrointestinal, cardiovascular, and genitourinary tracts. Pulmonary disease is the source of over 90% of the morbidity and mortality of these patients [2]. In pregnancy, additional changes affect the lungs. The enlarged uterus displaces the diaphragm upwards, which decreases total lung capacity and residual volume. Cardiac output, blood volume, and minute ventilation increase [3]. One of the most important goals in anesthetic management is to prevent respiratory decompensation since this is the most common cause of death [2].

This case report describes a 21-year-old parturient with CF who presented with multiple predictors of a poor outcome including a severely decreased FEV1, frequent pulmonary infections, diabetes mellitus, and pancreatic insufficiency [2], undergoing cesarean delivery. It examines the cardiovascular implications of this increasing patient population focusing on perioperative ECMO use and backup noninvasive ventilation [4]. Written consent for this report has been obtained from the patient.

## 2. Case Report

A 21-year-old primigravida with cystic fibrosis presented for elective cesarean delivery at 34 weeks' gestation. Before becoming pregnant, she suffered recurrent chest infections requiring two to four courses of intravenous (IV) antibiotics per year and her sputum was chronically colonized with *Pseudomonas aeruginosa*, *Stenotrophomonas*, and MRSA. Her comorbidities included Class A1 diabetes mellitus, pancreatic insufficiency, and chronic hypoxemic respiratory failure on chronic prednisone 2.5 mg daily, requiring 4 L/min home oxygen at night with nasal cannula (NC). Throughout pregnancy, she required additional oxygen during the day at 2–3 L/min. Her baseline FEV1 was 27% of predicted. She denied any history of cardiac disease. She met lung transplantation criteria due to FEV1 <30% and recurrent exacerbations requiring IV antibiotics, which would be pursued following her delivery.

She was counselled at 11 weeks' gestation regarding the risk of serious worsening of her lung function but decided to continue with the pregnancy. At 24 weeks' gestation, she was admitted with deteriorating respiratory function (forced expiratory volume in one second (FEV1) 0.64 L, 21% of predicted) and shortness of breath. She was treated with IV antibiotics and intensive chest physiotherapy. On discharge, fifteen days later, her lung function had returned to baseline.

A multidisciplinary meeting was organized with maternal-fetal medicine, obstetric anesthesiology, pulmonology, MICU, cardiac anesthesiology, cardiothoracic surgery, and NICU. Due to her poor lung function, pulmonology advised against normal vaginal delivery. The anesthetic plan was to prevent pulmonary decompensation and to avoid intubation because of the high likelihood of prolonged mechanical ventilation postoperatively. The patient would receive neuraxial anesthesia with general anesthesia as backup. A cardiac anesthesiologist would be available to assist with management and to perform intraoperative transthoracic or transesophageal echocardiogram if needed. Preemptive bilateral femoral sheath placement in preparation for possible cannulation for venovenous extracorporeal membrane oxygenation (ECMO) was also planned in case of intraoperative or postoperative decompensation. A low Pfannenstiel incision was planned and the MICU team arranged for postoperative ICU placement for close monitoring.

A cesarean section was scheduled at 34 weeks of gestation, to avoid further deterioration in her lung function, because fetal growth began to slowdown and the mother started to lose weight. She was admitted six days before surgery weighing 53 kg. She received dexamethasone for fetal lung maturation, and a routine echocardiogram was performed at this stage which showed no evidence of pulmonary hypertension. Her diabetes was well controlled with insulin sliding scale. Chest physiotherapy continued 3 times a day. She was on amoxicillin/clavulanate for positive *H. influenza* sputum cultures, and she was switched to cefepime and aztreonam on admission and continued her home nebulization treatments with albuterol, dornase alfa, and acetylcysteine.

On the day of surgery, she received her bronchodilators and mucolytics and she was transferred to the OR after her hyperreactive airway symptoms subsided following chest physiotherapy. She was also premedicated with 30 mL of oral sodium citrate and 10 mg of IV metoclopramide. In the OR, a BiPAP machine, a high flow nasal cannula, and a bronchoscopy cart were readily available.

Monitoring consisted of pulse oximetry, electrocardiogram, capnography, and direct intra-arterial blood pressure measurement after an arterial line was placed. Two 18-gauge peripheral cannulas were sited intravenously. A modified combined spinal-epidural was performed in the sitting position at L4-L5, with 9 mg of bupivacaine 0.75%, 15 mcg of fentanyl, and 100 mcg of preservative-free morphine given intrathecally. An epidural catheter was left in place. Only 9 mg of bupivacaine was administered aiming to slowly build a T5 sensory level with epidural medication and start placement of femoral sheaths simultaneously. Instead, the patient quickly developed a T2 sensory level but had nonlabored breathing on NC at 4 L/min and tolerated the anesthesia well. Phenylephrine (50 mcg/mL) infusion was started after injecting the intrathecal medications at a rate of 20 mcg/kg/min and titrated to keep MAP above 90. The patient was positioned laying on a wedge pillow, with her back up 30 degrees, because she could not tolerate lying supine. Oxygen was continued via a NC 4 L/min, and oxygen saturations were maintained above 95%. Arterial blood gas obtained after supine position showed pO_2_ of 104 mmHg, no acid-base imbalances, and hematocrit of 31%.

Bilateral femoral veins were cannulated with 16-gauge introducer sheaths, which would allow for rapid cannulation for ECMO if necessary. The patient received 2 g of cefazolin IV for surgical prophylaxis, and surgery was started. Shortly after incision, a female neonate was delivered with Apgar scores of 9 at 1, 5, and 10 minutes. Oxytocin infusion was started immediately after delivery and continued during the case. 15 minutes following delivery, the patient's oxygen saturation dropped to low 90 s and a Venturi mask at 10 L/min was temporarily required to improve her oxygen saturation, in addition to elevating the back of the bed. The patient received 1 g of acetaminophen and 30 mg of ketorolac IV as part of a multimodal pain regimen, and bilateral transversus abdominis plane (TAP) blocks with 266 mg of liposomal bupivacaine were performed in the OR following the end of surgery. Estimated blood loss was 600 mL. A final arterial blood gas again showed no acid-base disorders, pO_2_ was 114 mmHg, and hematocrit was 29%. The epidural catheter was removed at the end of the case, and the patient was transferred in stable condition on 4 L NC to a medical ICU for close monitoring.

She was placed on scheduled IV acetaminophen and ketorolac and reported excellent pain control with no supplemental opioids. Chest physiotherapy was resumed the same day. On postoperative day 1, the femoral sheaths were removed, subcutaneous heparin was started for deep venous thrombosis prophylaxis, the patient was ambulating, and she was transferred out of the ICU. While she remained inpatient, she continued to report significant improvement of the dyspnea. On the fifth postoperative day, she had no oxygen requirements on a six-minute walk test and her chest X-ray was stable, and on the following day, she was discharged home.

## 3. Discussion

Improved prognosis for CF patients means more women are surviving into their reproductive years. Both CF and pregnancy provide interesting physiologic and pathologic conditions. These changes negatively affect patients depending on several factors that predict poor outcome. The most reliable predictor is prepregnancy FEV1 [3, 5]. Although successful delivery is possible in a patient with the baseline FEV1 of 17% predicted [6], CF parturients with baseline FEV1 <60% are at higher risk for complications [3]. Other significant factors include frequent pulmonary infections, especially with *Burkholderia cepacia*, BMI <18 kg/m^2^, poor weight gain, pancreatic insufficiency, CF-related diabetes, pulmonary hypertension, and cor pulmonale [2, 3, 5]. These factors together may predispose patients to the most common cause of morbidity and mortality and respiratory deterioration [2, 7]. In addition to that, the increase in circulating blood volume and cardiac output that occur in pregnancy may lead to right heart strain and right heart failure in the setting of moderate to severe pulmonary disease [8].

Pregnant CF patients who may not tolerate the respiratory demands of labor or reaching full term may require scheduled cesarean section [2]. The choice for timing of delivery should balance the risks of respiratory complications to the mother and the risks of prematurity to the baby [9]. This decision should involve the mother and a multidisciplinary team. These patients should be managed in a tertiary center during the perinatal period by a maternal-fetal medicine team in consultation with pulmonology, cardiology, respiratory therapy, obstetric anesthesiology, and NICU for optimal care. Management of diabetes and nutritional deficiency, optimization of respiratory function, and management as well as prophylaxis of pulmonary exacerbations should be addressed during the prenatal period [10].

Among the cardiac complications in CF, cor pulmonale and pulmonary hypertension are the most concerning. Even pediatric CF patients have early signs of potential cardiac impairment, and in the Adult population, changes can be found in the left and right ventricular function. Inflammation and hypoxemia seem to play an important role in the development of these changes [11, 12]. Several studies suggest that cor pulmonale and pulmonary hypertension are absolute contraindications for pregnancy in CF women [4, 13]. Increased pressure in the right ventricle can cause right ventricular dysfunction, tricuspid regurgitation, and, in severe cases, death. Women with pulmonary hypertension before pregnancy may also have right ventricular hypertrophy or arrhythmias [14]. Strong data specifically linking cor pulmonale to poor outcomes in CF pregnant women could not be located. However, the mortality in parturients with pulmonary hypertension is around 30% [14–16]. A systematic review reported a mortality rate of 17–33% and recommended discussing termination with patients if pregnancy occurs [16]. Screening patients with CF with an echocardiogram during prenatal care is very important. This patient fortunately had a normal echocardiogram.

The common goal in the anesthetic care of all CF patients is to avoid pulmonary deterioration [2]. Regardless of the anesthetic technique, these patients should have recent pulmonary function tests and they should receive their usual treatment with inhalers and mucolytics as well as chest physical therapy in the preoperative period. Premedication with antacid and/or H_2_ blockers and prokinetic agents should be considered since gastroesophageal reflux is common in pregnancy and aspiration would be catastrophic in a CF patient.

General anesthesia should be avoided when possible due to its potential to worsen lung function [16]. CF patients are more prone to have obstructive physiology with air trapping and intrinsic PEEP, and they are at risk for barotrauma, bronchospasm, and pneumothorax with invasive mechanical ventilation (IMV) [17]. In the presence of cor pulmonale, positive pressure ventilation during induction and maintenance of anesthesia can have serious deleterious effects in hemodynamics. Clearance of secretions would also be a challenge during IMV in CF. In addition to that, IMV in the setting of acute respiratory failure in a CF patient could result in chronic dependency of mechanical ventilation. For all these reasons, neuraxial anesthesia was chosen for our patient.

In this case, a modified combined spinal-epidural neuraxial technique was preferred for slow titration of the anesthetic level. A goal sensory level of T6 instead of the routine goal of T4 was chosen due to its success in previous cases and in order to minimize the possible impact in intercostal musculature and thus in the ventilation dynamics [7, 18]. This anesthesia technique also had the advantage of allowing for administration of neuraxial morphine. Despite low dose of intrathecal hyperbaric bupivacaine, this patient developed a T2 level to cold, but her respiratory function did not significantly deteriorate. The patient was kept in a semi-sitting position to additionally increase respiratory compliance. Strategies to avoid intubation included having humidified Venturi mask, high-flow nasal cannula, and BiPAP available as alternatives to escalate noninvasive ventilation (NIMV). A bronchoscopy cart was also available for management of lower airway obstruction with secretions after intubation and IMV if needed. Arterial line placement should be considered for either neuraxial or general anesthesia for continuous monitoring and for frequent arterial blood gases. Having a cardiac anesthesiologist available to assist with management and with transesophageal echocardiogram if significant intraoperative clinical deterioration may be helpful.

Extracorporeal membrane oxygenation (ECMO) can be a strategy used as a bridge to lung transplant or to recovery in a carefully selected CF population. ECMO continues to be considered a relative contraindication for lung transplant in CF patients [19]; however, there have been technical advances in this area and positive outcomes [20]. A retrospective review of the Extracorporeal Life Support Organization (ESLO) Registry reported that 52% of CF patients survived their ECMO run and there was no difference in survival when venovenous (VV) and venoarterial (VA) ECMO were compared [20, 21]. ECMO use in CF patients is more often discussed as a controversial bridge to transplantation when ventilatory support is insufficient [17, 20–23]. The respiratory indications for transplantation in CF are severe respiratory disease with FEV1 <30%, type 1 or 2 respiratory failure, and recurrent pneumothoraces or hemoptysis not responsive to treatment [23]. A retrospective analysis of the United Network for Organ Sharing database suggested that patients on ECMO with spontaneous breathing had improved survival compared with other bridging strategies [24]. ECMO is also associated with possible serious complications such as hemorrhage, vascular rupture, infection, and failure of other organs, and some studies argue that these risks and the high mortality rate associated with ECMO outweigh its benefits [20, 21].

The data on use of ECMO for the obstetric population with CF are extremely limited. These patients may present rapid deterioration due to exacerbations of their baseline pathology combined with the expected physiologic changes inherent to the peripartum period, and ECMO may play an important role in the acute respiratory failure phase. The selection of the type of ECMO should be considered ahead of time. VV ECMO could potentially be a better option if the need for ECMO is pure ventilatory secondary to progression of pulmonary disease. On the other hand, VA ECMO can be beneficial when cor pulmonale is present. In our case, the possibility of an emergency lung transplant was thoroughly discussed with the patient and cardiothoracic surgery was consulted to participate in her care. In this scenario, bilateral femoral venous sheaths were placed after neuraxial procedure and prior to starting surgery in preparation for potential cannulation and VV ECMO initiation. It was determined that this approach was less invasive than preemptive cannulation while providing access, so cannulation could be performed rapidly if needed.

Pain control is a major concern in the peripartum period of a CF patient. These patients should have optimal analgesia in order to continue chest physical therapy, to be able to cough and clear secretions, to maintain their respiratory dynamics, and to be able to mobilize early. A multimodal pain regimen as an opioid sparing strategy should be applied to reduce the risk of opioid-induced respiratory depression [2, 17]. Our patient received low-dose intrathecal morphine as it is the gold standard option for postcesarean analgesia with the benefit of providing less sedation and lower early postoperative total opioid consumption than systemic opioids [25]. Intraoperatively she received acetaminophen and ketorolac, and immediately after surgery she received bilateral TAP blocks with liposomal bupivacaine with the goal of prolonged postoperative analgesia and decreased overall opioid consumption. Using the epidural catheter would also be a good option as part of the regimen for postoperative analgesia, but this was not logistically feasible in our institution.

These patients should be closely monitored in the early postoperative phase in an ICU with availability of NIMV and IMV [18]. Maintenance of their respiratory treatment regimen, antibiotic regimen for treatment, or prophylaxis of infections as well as pain control and thromboprophylaxis are very important considerations. Our patient was immediately transferred to a medical ICU where she was followed by all the teams involved in her preoperative care, she maintained good saturations, she continued chest physical therapy with no problems, and she reported significant improvement of the dyspnea as well as excellent pain control with no supplemental opioids. She was discharged from the ICU on postoperative day 1, and she was later able to go home with no complications.

## 4. Conclusion

CF is a multifaceted, chronic, and life-threatening disease. With the advances in therapy, more CF patients are living until reproductive age, but the mortality remains very high [22]. Our case highlights the value of multidisciplinary management with optimization of the patient as well as thorough planning to prevent and manage complications and improve outcomes, in addition to screening for cardiovascular changes and the considerations for ECMO as a backup plan along with the limitations in the literature showing a need for further research in the area.

## 5. Authors' Contribution

Thais Franklin Dos Santos helped manage the patient, conduct the background research, and write the manuscript. Andrea Rabassa helped manage the patient, conduct the background research, and write the manuscript. Oscar Aljure helped write and edit manuscript. Reine Zbeidy helped manage the patient, conduct the background research, and write the manuscript.

## Additional Points

Cystic fibrosis is associated with multiple organ systems being affected including the respiratory, gastrointestinal, cardiovascular, and genitourinary tracts. Anesthetic challenges augmented in pregnant patients with cystic fibrosis. Multidisciplinary planning is needed for successful outcome in these patients.
